# Characteristics of SARS-CoV-2 Delta variant-infected individuals with intermittently positive retest viral RNA after discharge

**DOI:** 10.1093/nsr/nwac141

**Published:** 2022-07-26

**Authors:** Lu Li, Jingyan Tang, Zhiwei Xie, Qingxin Gan, Guofang Tang, Zhongwei Hu, Huimin Zeng, Jingrong Shi, Jiaojiao Li, Yan Li, Changwen Ke, Min Kang, Dan Liang, Huan Lu, Yuwei Tong, Xilong Deng, Jinxin Liu, Hongzhou Lu, Fuxiang Wang, Fengyu Hu, Feng Li, Nanshan Zhong, Xiaoping Tang

**Affiliations:** Guangzhou Eighth People's Hospital, Guangzhou Medical University, China; Guangzhou Eighth People's Hospital, Guangzhou Medical University, China; Guangzhou Eighth People's Hospital, Guangzhou Medical University, China; Guangzhou Eighth People's Hospital, Guangzhou Medical University, China; Guangzhou Eighth People's Hospital, Guangzhou Medical University, China; Guangzhou Eighth People's Hospital, Guangzhou Medical University, China; Guangzhou Eighth People's Hospital, Guangzhou Medical University, China; Guangzhou Eighth People's Hospital, Guangzhou Medical University, China; Guangzhou Eighth People's Hospital, Guangzhou Medical University, China; Guangdong Provincial Center for Disease Control and Prevention, China; Guangdong Provincial Center for Disease Control and Prevention, China; Guangdong Provincial Center for Disease Control and Prevention, China; Guangdong Provincial Center for Disease Control and Prevention, China; Guangzhou Eighth People's Hospital, Guangzhou Medical University, China; Guangzhou Eighth People's Hospital, Guangzhou Medical University, China; Guangzhou Eighth People's Hospital, Guangzhou Medical University, China; Guangzhou Eighth People's Hospital, Guangzhou Medical University, China; The Third People's Hospital of Shenzhen, China; The Third People's Hospital of Shenzhen, China; Guangzhou Eighth People's Hospital, Guangzhou Medical University, China; Guangzhou Eighth People's Hospital, Guangzhou Medical University, China; Guangzhou Laboratory, Bio-Island, China; National Clinical Research Center for Respiratory Diseases of First Affiliated Hospital Guangzhou Medical University, China; Guangzhou Laboratory, Bio-Island, China; Guangzhou Eighth People's Hospital, Guangzhou Medical University, China; Guangzhou Laboratory, Bio-Island, China

SARS-CoV-2-infected patients will be discharged when viral ribonucleic acid (RNA) is consecutively negative at least twice over a 24-h interval. However, we have demonstrated that positive retest viral RNA, a particular clinical phenomenon initially observed in discharged SARS-CoV-2-infected patients in early 2020 [1,2], is the occasional reoccurrence of Polymerase Chain Reaction (PCR)-detectable viral RNA but not reinfection [3]. Unfortunately, workable strategies to effectively discriminate against individuals likely to have positive retest viral RNA are still absent. Thus, for safety concerns, a mandatory minimum of 14-day quarantine in hospitals or hotels had been strictly applied for discharged patients. Given that the Delta Variant of Concern (VOC), the dominant SARS-CoV-2 strain in 2021, becomes more infectious and produces ∼1000-fold higher viral titers compared to the ancestral strain [4], more strict quarantine management has been applied for all discharged Delta-infected patients. However, whether robust viral replication leads to severe viral lingering in Delta variant-infected individuals remains less investigated.

This retrospective study provides comprehensive characteristics of the positive retest Delta virus by including nearly all the Delta variant-infected individuals admitted to Guangzhou Eighth People's Hospital, Guangzhou Medical University. Our analysis revealed that 77 of 158 (48.73%) local and 437 of 679 (64.36%) imported individuals infected with SARS-CoV-2 Delta variant still had positive retest viral RNA (Fig. 1A), significantly exceeding what happened to the early viral strain in 2020 (7.2%) [3]. For local patients with positive retest viral RNA, 95% and 45% of the patients required another 21 and 42 days, respectively, to become completely viral RNA negative (Supplementary Fig. S1A). For imported patients, 85% and 23% of them required another 14 and 21 days, respectively, to completely clear their viral residues (Supplementary Fig. S1B). Our observation highlighted that the Delta viral infection caused a substantially longer viral RNA persistence and their viral RNA shedding *in vivo*, combining the first administration and the intermittent positive retest, was significantly extended to >5 months in some individuals (Fig. 1B).

**Figure 1.**
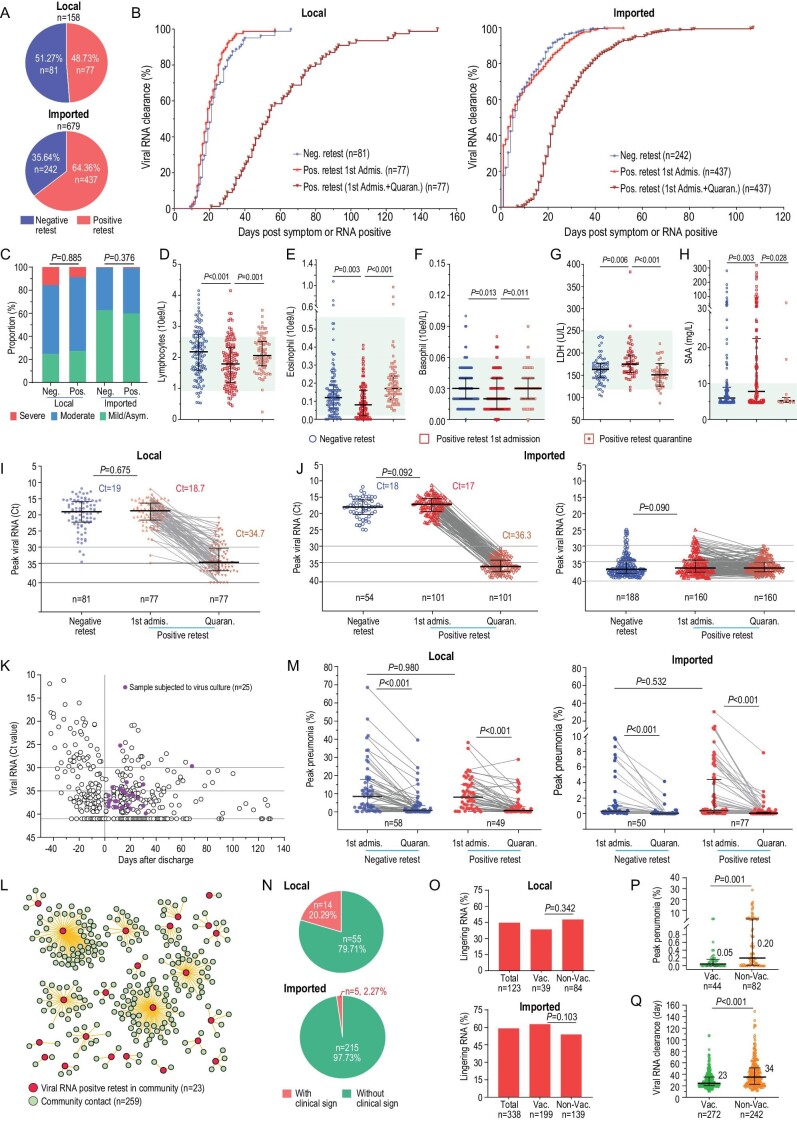
Delta-variant-infected patients with positive retest viral RNA after discharge. (A) The proportion of patients with positive retest viral RNA in local and imported COVID-19 patients. Case number and percentage are shown. (B) Kinetics of SARS-CoV-2 RNA clearance in patients with positive retest and negative retest viral RNA in local and imported patients. Blue and red: virus RNA clearance of negative and positive retest patients during the first admission period, respectively. Brick red: virus RNA clearance of positive retest patients during the first admission plus quarantine period. (C) Disease severity with occurrence of positive retest viral RNA. Symptoms are indicated. (D)–(F) Different blood-cell counts in patients with positive retest viral RNA. (D) Lymphocytes. (E) Eosinophils. (F) Basophils. (G) and (H) Different laboratory tests in patients with positive retest viral RNA. (G) Lactate dehydrogenase (LDH) test. (H) Serum amyloid A (SAA) test. (I) Peak viral RNA during first admission and subsequent quarantines of local patients. (J) Peak viral RNA during first admission and quarantine of imported patients. Left, peak viral RNA Ct < 25; right, peak viral RNA Ct > 25. One hundred and sixty-two patients who tested negative during the first admission and 14 patients who had positive retest viral RNA with titers Ct < 30 during quarantine are excluded for a separate analysis (Supplementary Figs S4 and S5). (K) Alive virus culture from nasopharyngeal swabs with positive retest viral RNA collected during quarantine. Twenty-five samples (purple) obtained form 23 patients were used for alive virus culture. Viral titers (Ct) are plotted. (L) Epidemiological analysis of the transmission risk of patients with positive retest viral RNA to their close contacts in the community. Red circle: patients with positive retest viral RNA; green circle: close contacts in the community. (M) Lung damage recovery assessment for local and imported patients with positive retest viral RNA. Each point represents the peak volume ratio of lung lesion for each case. (N) Frequency of clinical signs among patients with positive retest viral RNA during quarantine. Clinical signs included cough, expectoration, throat discomfort, and tiredness (details see Supplementary Fig. S7). These clinical signs do not require medical treatment. (O) Assessment of vaccination on the ratio of viral RNA reoccurrence among local and imported patients. (P) Peak viral pneumonia between the non-vaccinated group and vaccinated group in positive retest patients. (Q) Protective effects of vaccination on viral RNA persistence in positive retest patients. Student's *t*-test was used in (I) and (J). Mann-Whitney *U* test was used to compare the blood-cell counts, laboratory tests and peak pneumonia between negative and positive retest patients during the first admission (D)–(H), (M) and in (P) and (Q). Wilcoxon matched-pairs test was used to compare the blood-cell counts, laboratory tests and peak pneumonia during first admission and in quarantine (D)–(H), (M). Chi-square test was used in (C) and (O). Neg., negative retest viral RNA group; Pos., positive retest viral RNA group, with positive retest viral RNA after discharge; 1st Admis., during the first admission period; Quaran., during the quarantine after discharge. Median and quartile are shown. Vac., vaccinated; Non-Vac., none vaccinated.

To efficiently discriminate those more likely to become viral RNA retest positive after discharge, we examined factors that might be associated with positive retest viral RNA. Demographic factors such as age, gender, symptoms, and most underlying diseases are unrelated to viral RNA reoccurrence (Supplementary Table S1). Patients with worsened clinical manifestations did not have a high frequency of positive retest viral RNA (Fig. 1C). Critical patients show a tendency to have a viral reoccurrence, but not reaching significance (Supplementary Table S1). Interestingly, we observed that the positive retest group tends to have lower serum lymphocytes, eosinophils, and basophils (Fig. 1D–F and Supplementary Table S2, but still within normal ranges) and increased serum lactate dehydrogenase (LDH) and serum amyloid A (SAA) (Fig. 1G)and H and Supplementary Table S2, still within normal ranges) on admission. Finally, we found that the peak viral titers had no difference (Fig. 1I)and J), which phenomena were similar to the early SARS-CoV-2 virus in 2020 [3], despite the fact that the Delta variant infection produces ∼1000-fold more virus than ancestral strains [4]. Therefore, demographic factors and primary clinical tests cannot serve as workable biomarkers in practice to predict viral RNA reoccurrence.

The local outbreak of Delta infection in Guangzhou in 2021 provided a unique opportunity to longitudinally investigate the full spectrum of viral kinetics in the Delta-infected patients from early infection, to discharge, to quarantine, and even after they were released into the community [4]. We observed that positive retest viral RNA titers were

∼10e5- to 10e6-fold (16- to 19-Ct cycles) lower compared to their first hospitalization (Fig. 1I). The imported patients were a little complicated because a large portion of imported patients were PCR-confirmed and admitted to the hospital during their late stage of viral infection (Ct > 25, 160 cases) or after the viral clearance (viral RNA negative, 162 cases) (Supplementary Fig. S2A). Subgroup analysis based on the viral titers revealed a similar declining tendency among imported patients (19-Ct number decline) to local patients (Fig. 1J). Notably, 162 imported cases with no detectable virus during the first hospitalization had positive retest viral RNA during the subsequent quarantine isolation, but their peak virus titers were over Ct > 30 (Supplementary Fig. S2B).

From an epidemiological perspective, only samples with the alive virus are potentially contagious and pose a public health risk. One major challenge is that the current SARS-CoV-2 qRT-PCR quantification cannot discriminate infectious viral particles from non-infectious viral RNA. For the Delta variant, the non-infectious viral RNA fragments outnumbered the infectious vial particles by 10e5–10e8 times in nasopharyngeal samples [5]. Then, we divided the patients based on the maximal viral titers into Ct < 30, 30–35 and ≥35 groups. Combining the local and imported individuals with positive retest viral RNA, 481 of 514 (93.6%) individuals had Ct ≥ 30 (Supplementary Fig. S3A–C). Only 19 local and 14 imported patients of a total of 514 (6.42%) individuals had Ct values of <30 (Supplementary Fig. S3D). Interestingly, the residual viral RNA showed up intermittently for most individuals and high viral titers were seldomly detected (Supplementary Figs S4 and S5), also supporting that those patients are less likely to be of a consistent transmission origin. Moreover, non-vaccination status, old age, comorbidity, and severe symptoms were found to be associated with the seldom high levels of positive retest viral RNA (Supplementary Fig. S6). Since most of the population have received the full course of vaccination, a combination of non-vaccination, old age, and underlying diseases can serve as prognostics for discriminating those patients who are likely to have high concentrations of reoccurred viral RNA.

Next, attempts to culture the alive virus from the available 25 upper respiratory samples using Vero-E6 cell culture methods failed, despite the Ct values of some samples being 29 and 25 (Fig. 1K). Additionally, we found that 23 cases with positive retest viral RNA in the community, who had had epidemiologically close contact with 259 individuals, caused no community transmission event (Fig. 1L). Our analysis supported the conclusion that individuals with positive retest viral RNA were less likely to spread the virus and were safe for the community.

Meanwhile, we evaluated the necessity for individuals to receive medical treatment if getting a positive retest after discharge. First, blood test result indicated that the abnormal cell counts of lymphocytes, eosinophils, and basophils and the elevated LDH and SAA levels returned to normal ranges (Fig. 1D–H). Second, positive retest viral RNA did not exacerbate lung damage on average (Supplementary Fig. S7A). Nevertheless, viral pneumonia was further absorbed during the quarantine except for some severe and critical cases (Fig. 1M)and Supplementary Fig. S7B–C). Our study revealed that health conditions improved despite residual viral RNA becoming detectable. Finally, only 19 of 289 (6.57%) patients showed recordable clinical symptoms such as occasionally coughing, expectoration, throat discomfort, and tiredness when viral RNA was retested (Fig. 1N)and Supplementary Fig. S7D–E). In short, we demonstrated that in-hospital treatment is unnecessary for most patients even with positive retest viral RNA except those who require medical care for other reasons, but not SARS-CoV-2 infection, since they have just survived severe or critical periods.

Furthermore, to better understand the protective effects of vaccination, we compared whether viral RNA still lingered in the vaccinated individuals because breakthrough infection frequently occurred for Delta variants. Unfortunately, prior vaccination failed to reduce the occurrence of viral lingering (Fig. 1O). Encouragingly, vaccination significantly mitigated viral pneumonia in the presentence of positive retest viral RNA (Fig. 1P) and substantially shortened the viral RNA duration (Fig. 1Q).

In summary, our investigation revealed that positive retest SARS-CoV-2 viral RNA after discharge is less likely to be infectious. First, the viral RNA copies detected by qRT-PCR far exceed the infectious particles at ∼10e5–10e8 times [5–7]. When converted into Ct values, Ct = 30, equaling 10e5 copies/mL, could be a reasonable cut-off for infectiousness discrimination. The titers of positive retest viral RNA in 94% of the individuals was Ct > 30; thus, it was safe for the public in this regard. Second, reoccurred viral RNA rebound to higher concentration (Ct < 30) was observed seldomly; however, no alive virus was cultured from samples with positive retest viral RNA, despite one sample with Ct = 25. Meanwhile, a high concentration of viral RNA pops up intermittently and randomly, suggesting that this population will be safe most of the time. Third, the duration of infectious virus shedding in the upper respiratory tract was mainly within 2 weeks and less likely to be ≤3 weeks [5,7,8]. Even with the same viral RNA concentration, samples collected in the early viral increase phase were easier to get alive virus from than samples collected in the late viral declining phase [5]. Positive retest viral RNA was often detected >3 weeks after admission (Supplementary Fig. S1), far beyond the infectious episode. Finally, thanks to the stringent community follow-up management of the local Delta-infected individuals, we had the extreme opportunity to observe that lingering viral RNA could be retested positive monthly after discharge (Fig. 1B)and Supplementary Fig. S1) [3]. Based on this fact, some of the ‘recovered’ imported individuals will theoretically become retest positive in the community or workplace, which is unfortunately missed for multiple reasons. No community transmission ever reported corroborates the conclusion that individuals with positive retest viral RNA are safe.

## DATA AVAILABILITY

The data that support the findings of this study are available from the corresponding author (F.L., X.T.) upon reasonable request and with permission of Guangzhou Eighth People's Hospital, Guangzhou Medical University, Guangzhou, China.

## Supplementary Material

nwac141_Supplemental_file
