# Supplementary material for: Characteristics of SARS-CoV-2 Delta variant-infected individuals with intermittently positive retest viral RNA after discharge
Source: Natl Sci Rev. 2022 Jul 26;9(10):nwac141. doi: 10.1093/nsr/nwac141 (PMC9616468; doi:10.1093/nsr/nwac141)
Supplement: nwac141_Supplemental_file [file nwac141_supplemental_file.pdf]

Supplementary Information of

Title: Characteristics of SARS-CoV-2 Delta variant-infected individuals with intermittently positive retest viral RNA after discharge

This file contains:

Methods

Supplementary Figures 1-7

Supplementary Tables1-2

Methods

Patients Information

All patients in this study were from Guangzhou Eighth People's Hospital, Guangzhou Medical University. The local 158 patients from May 21 to Jun 18, 2021, were officially confirmed to be infected with SARS-CoV-2 Delta variant as previously reported[1]. Since the Delta variant became the dominant circulating SARS-CoV-2 variant worldwide from Jun to Nov, 2021, (https://nextstrain.org), we included all the 679 imported patients from Jul 1 to Nov 24, 2021, for analysis. Viral genomes from 178 patients were sequenced to be Delta variants. The rest patients were regarded as Delta-infected patients because their virus titers were too low for sequencing. This study was approved by Guangzhou Eighth People's Hospital Ethics Committee (No. 202001134 and 202115202). Written informed consents were obtained from all patients.

Criteria for Discharge and Definition of Re-test Positive

According to the Diagnosis and Treatment Protocol for Novel Coronavirus Pneumonia (Trial Version 8) published by the Chinese National Health Commission, patients with COVID－19 need to meet all the following criteria before hospital discharge: (a) normal

body temperature for more than 3 days, (b) significant improvement in respiratory symptoms, (c) substantial improvement in acute exudative lesions on chest computed tomography images, (d) two consecutive negative tests for SARS-CoV-2 RNA of respiratory specimens (sampling time interval of at least 24 hours). Moreover, patients who fulfilled discharge criteria were suggested to another 14-day quarantine to monitor their health. In this study, patients with detectable viral RNA during quarantine after discharge were defined as patients with positive retest viral RNA. Otherwise, they were defined as patients with negative retest viral RNA.

Data Collection

The demographic profiles, clinical characteristics, laboratory data, and chest imaging examinations for all COVID-19 patients were collected from the hospital information system and laboratory information system. COVID-19 severity classification includes four types: Mild cases refer to those who had mild clinical symptoms without signs of viral pneumonia on imaging. Moderate cases refer to those who had a fever, respiratory tract symptoms, and a sign of pneumonia on imaging. Severe cases refer to those who meet any of the following: (1) shortness of breath, respiratory rate≥30 times/min; (2) oxygen saturation≤93% in resting state; (3) arterial partial pressure of oxygen (PaO2)/inhaled oxygen concentration (FiO2) ≤300mmHg(1mmHg=0.133kPa); and (4) progressively worsening of clinical symptoms and progression of pneumonia lesions>50% within 24 to 48 hours on imaging. Critical cases refer to those who meet any of the following: (1) respiratory failure requiring mechanical ventilation; (2) shock; (3) combining with other organ failure requiring ICU monitoring and treatment. In addition, asymptomatic cases refer to those who test positive for SARS-CoV-2 viral RNA but have no clinical symptoms.

Viral RNA Detection with RT-PCR

The detailed protocol for sample collection and viral nucleic acid detection is as previously described[1, 2]. Briefly, the nasopharyngeal or oropharyngeal swabs were collected by trained medical staff according to standardized procedures. Viral RNA extraction and the subsequent RT-PCR detection were performed using the Nucleic Acid Isolation Kit (Da'an

Gene Co. Ltd, Cat: DA0630, China) and RNA Detection Kit (Da'an Gene Co. Ltd, Cat: DA0930, China) according to manufacturer's instructions. A positive test for SARS-CoV-2 was determined if a cycle threshold value (Ct value) was less than 40 either for N or ORF1a/b gene. All tests were performed according to standard operating procedures under strict biosafety conditions. The detection limit of cycle threshold (Ct) was set to be 40 (200 copies/ml).

Quantitative Analysis of Lung Lesions in Chest Computed Tomography

The lung lesions were calculated by an artificial intelligence (AI) model developed in Guangzhou Eighth People's Hospital as reported previously[3]. The chest CT imaging data set from SARS-VoV-2 viral RNA confirmed patients were collected from the hospital information system and were subject to two-step calculations. Briefly, the AI model would first detect any abnormality in every single slice and discriminate which lesion was caused by the viral infection; then, it summed up the value of the continuous lesion region to generate the volume ratio (VR) of the lesion in the left and the right lung. The volume of left lung (46.5%) and right lung (53.5%) were adjusted according to a previous study[4]. Total volume ratio (VR) = 46.5% * left VR + 53.5% * right VR. Experienced radiologists confirmed the calculations.

Epidemiological Investigation

Guangzhou local patients were regularly followed by the local center for disease prevention and control after return to the community. If they had detectable virus RNA, they were sent back to our hospital in time. Meanwhile, the close contacts with individuals with positive retest viral RNA samples were sampled for SARS-CoV-2 RT-PCR detection. They were closely monitored for possible transmission. All epidemiological data were recorded.

Alive virus culture experiment

The nasopharyngeal swabs with positive retest viral RNA were collected during

quarantine. The samples were collected in UTM-RT® tubes that can preserve alive viruses and were frozen in a -80℃ refrigerator. Then they were transported to BSL-3 laboratory by the cold chain for virus isolation and culture experiments. Vero-E6 cells, an epithelial continuous cell line from the kidney of a normal monkey (Cercopithecus aethiops), were available from GDCDC. Vero-E6 cells were cultured in the growth media (GM), Minimum Essential Medium (MEM) (GIBCO, Life, USA) supplemented with 1% 1M HEPES (GIBCO, Life, USA), 1% 100 IU/mL Penicillin Streptomycin (PS) (GIBCO, Life, USA) and 10% Fetal Bovine Serum (FBS) (GIBCO, Life, USA), and added into 3 mL inclined culture tubes at the final concentration of 1.5*10^5 cells per well, at 37℃, in a 5% CO2 incubator. After adherent sub-confluent cell monolayers of Vero-E6, the culture medium was refreshed, MEM containing 2% FBS, 1% HEPES, and 1% PS (constituting the maintenance media, MM) for the cell isolation of SARS-CoV-2 (200ul sample + 2mL MM per tube). The tubes were inspected for the appearance of cytopathic effect (CPE) every day during the 7-day experiment by an inverted microscope in certified Biosafety Level-3 Laboratory. After continuously propagating for 2 generations, and the results were recorded.

Statistics Analysis

All analyses were performed using SPSS software (IBMSPSS Statistics, version 25). Continuous variables were expressed as mean ± standard deviation (SD) or median (P25-P75), as appropriate. Categorical variables were summarized as the counts and percentages in each category. Two independent continuous variables were tested with a t-test for normal distribution and Mann-Whitney U test for abnormal distribution. Two paired quantitative variables were tested by paired t test or paired Wilcoxon test (for non-parametric data). The categorical variables were tested by Chi-square test. A value of $P<0.05$ was considered significant.

References

1. Wang Y, Chen R, Hu F, Lan Y, Yang Z, Zhan C*, et al.* Transmission, viral kinetics and

clinical characteristics of the emergent SARS-CoV-2 Delta VOC in Guangzhou, China. **EClinicalMedicine 2021**, 40**:** 101129.

2. Hu F, Chen F, Ou Z, Fan Q, Tan X, Wang Y*, et al.* A compromised specific humoral immune response against the SARS-CoV-2 receptor-binding domain is related to viral persistence and periodic shedding in the gastrointestinal tract. **Cell Mol Immunol 2020**, 17(11)**:** 1119-1125.
3. Yang Y, Lure FYM, Miao H, Zhang Z, Jaeger S, Liu J*, et al.* Using artificial intelligence to assist radiologists in distinguishing COVID-19 from other pulmonary infections. **J Xray Sci Technol 2021**, 29(1)**:** 1-17.
4. Yamada Y, Yamada M, Yokoyama Y, Tanabe A, Matsuoka S, Niijima Y*, et al.* Differences in Lung and Lobe Volumes between Supine and Standing Positions Scanned with Conventional and Newly Developed 320-Detector-Row Upright CT: Intra-Individual Comparison. **Respiration 2020**, 99(7)**:** 598-605.

Supp Figure 1

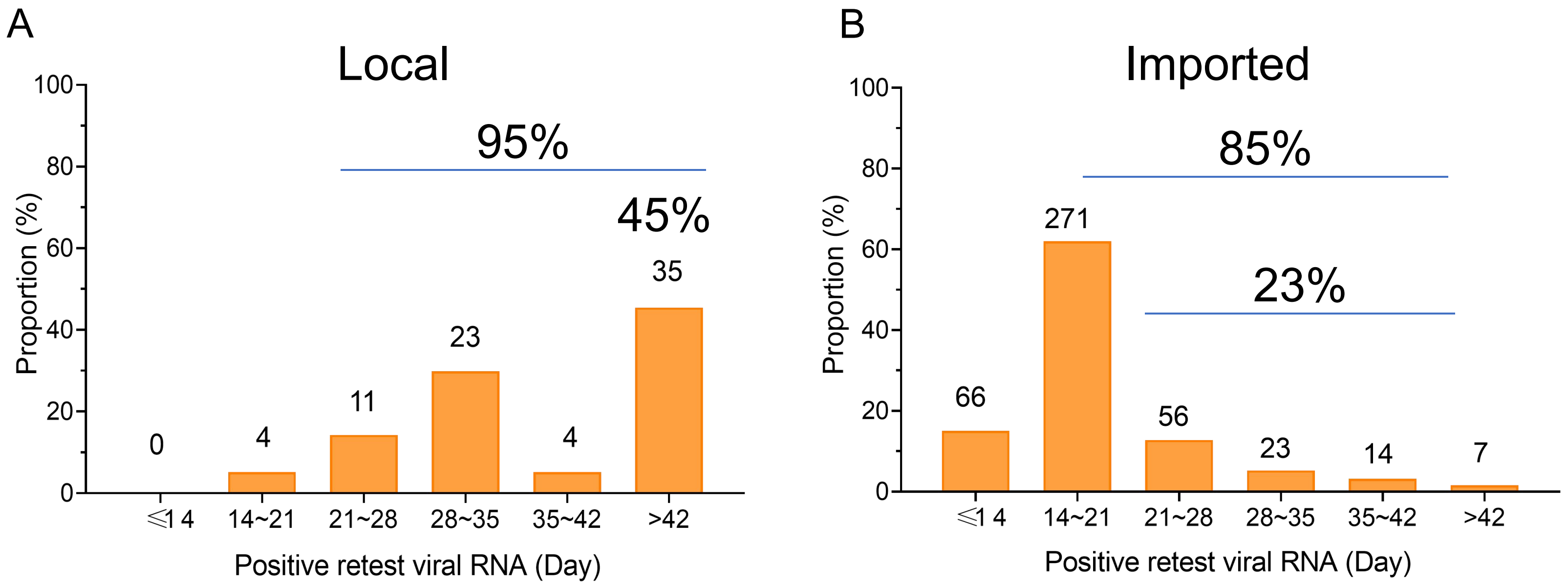

Supplementary Figure 1. Time required for positive retest viral RNA clearance. (A) Constitution analysis of viral RNA persistence for local Delta-infected patients. (B) Constitution analysis of viral RNA persistence for the imported Delta-infected patients.

Supp Figure 2

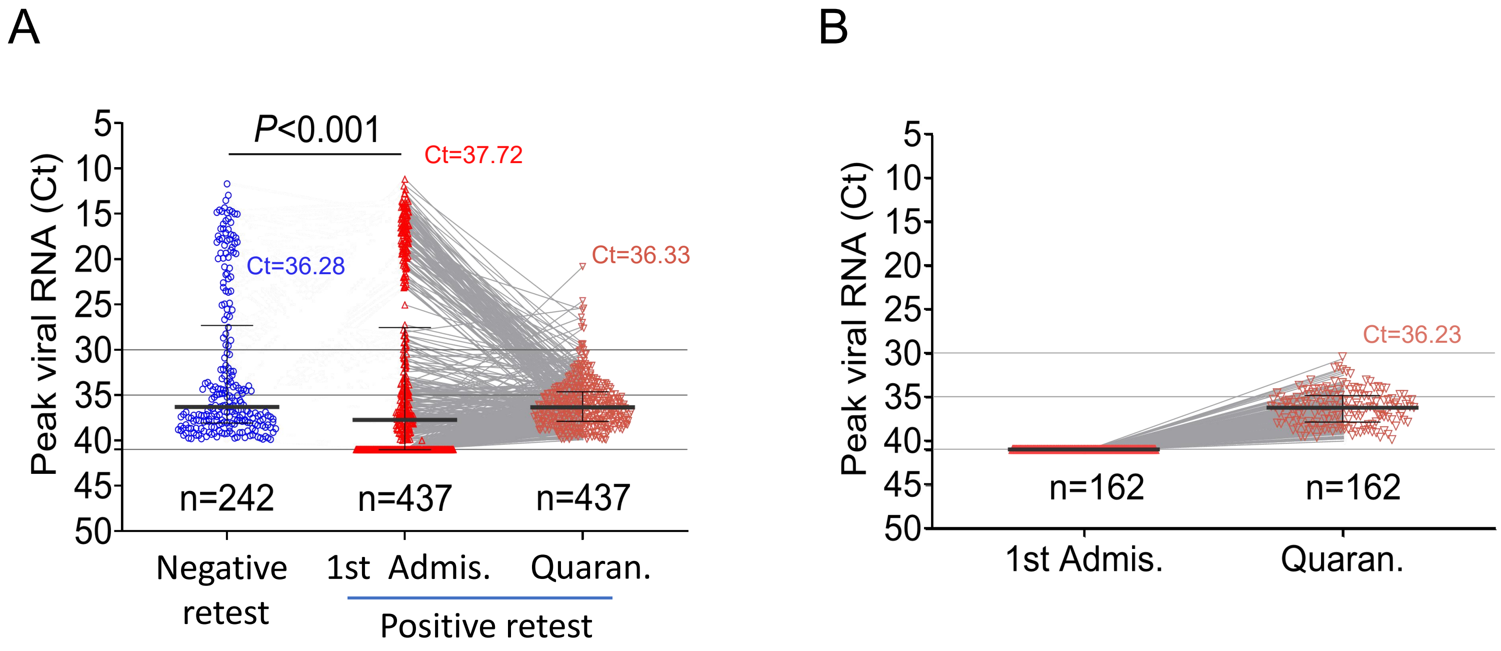

Supplementary Figure 2. Peak viral RNA during first admission and quarantine of imported patients. (A) Peak viral RNA titer analysis of 437 positive retest patients after discharge. (B) Peak viral RNA titers in 162 imported patients with undetectable viral RNA in the first hospital admission but with detectable viral RNA during quarantine. Blue, patients with undetectable viral RNA after discharge. Red, patients with positive retest viral RNA after discharge. Median is shown. *P* values are determined using the Mann-Whitney U test.

Supp Figure 3

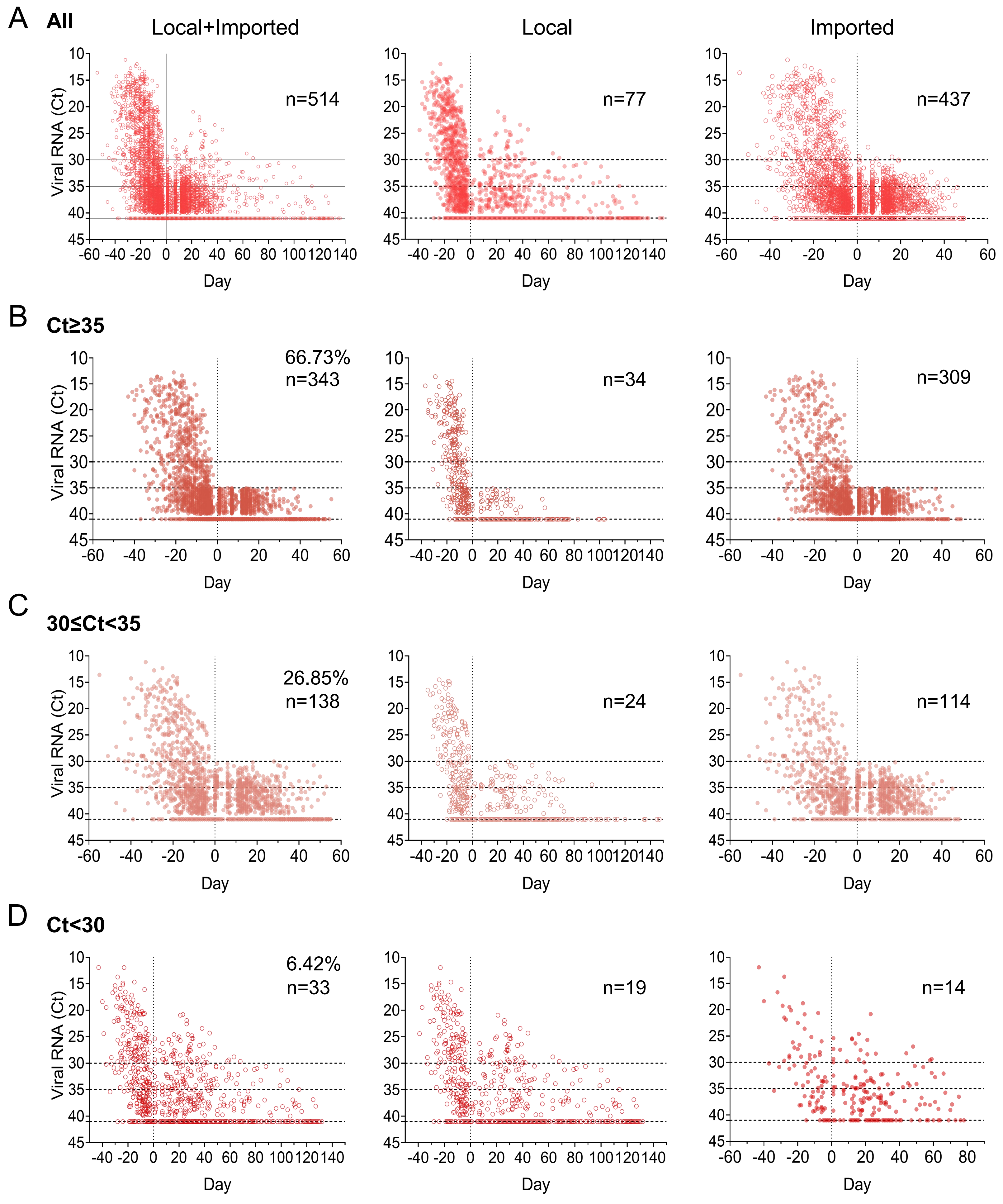

Supplementary Figure 3. Viral titers distribution of local and imported patients with positive retest viral RNA during quarantine. (A) Viral titers distribution of all local and imported patients with positive retest viral RNA . (B-D) Viral titers distribution in patients with low peak viral titer (Ct<35) (B), middle peak viral titer (30<Ct≤35)(C), and high peak viral titer (Ct≤30) (D) during quarantine.

Supp Figure 4

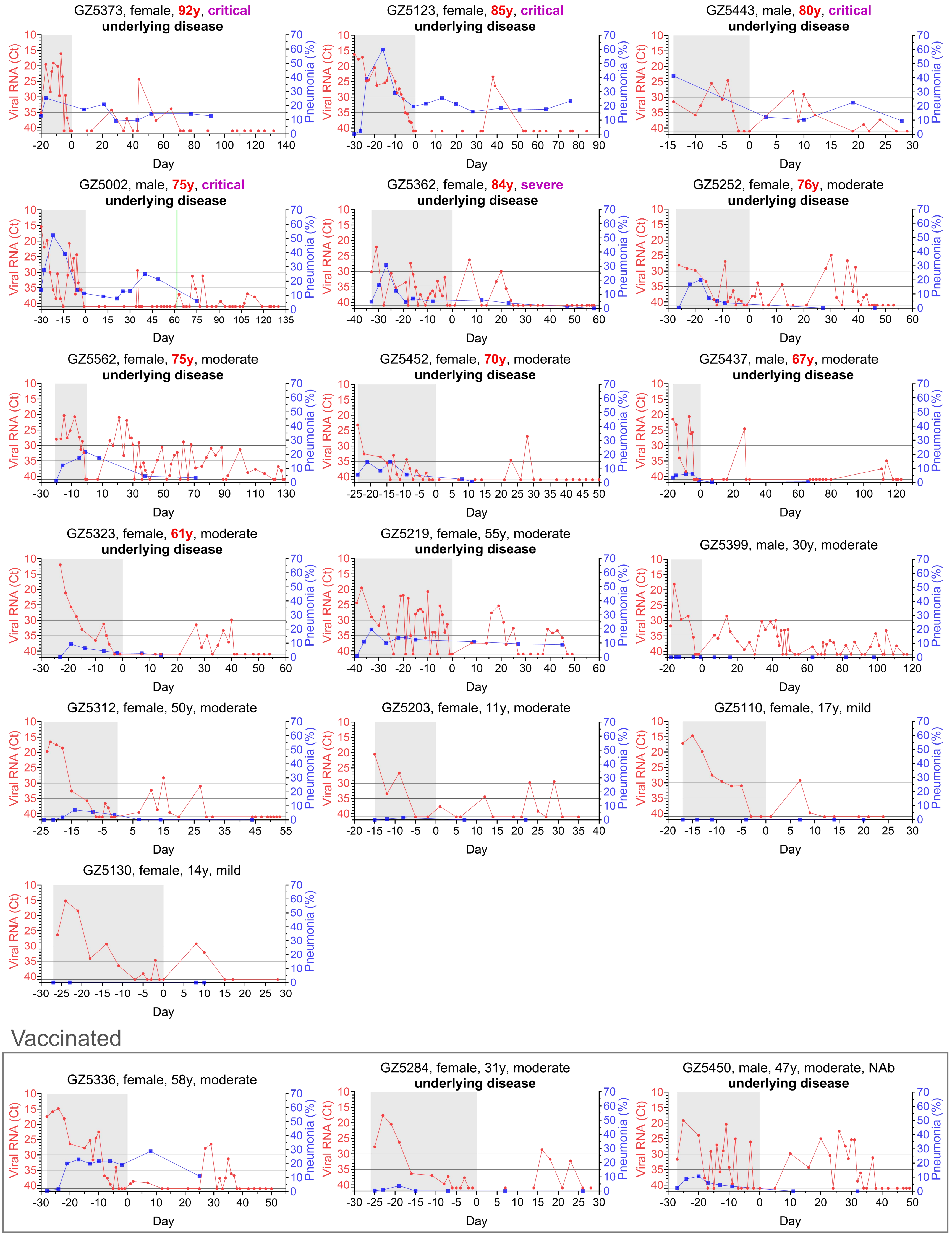

Supplementary Figure 4. Kinetics of viral RNA titers and pneumonia in 19 imported patients with a high retested viral RNA (at least one Ct<30) during quarantine. Patient number, gender, age, symptom, and comorbidity are labeled on the top of each individual. Red dot, viral RNA titers Ct value; blue square, pneumonia (ratio of abnormal lung lesions versus total lung volume); gray area, the first admission stage. Day 0 on the x-axis is the start time point when patients were discharged and transferred to quarantine wards.

Supp Figure 5

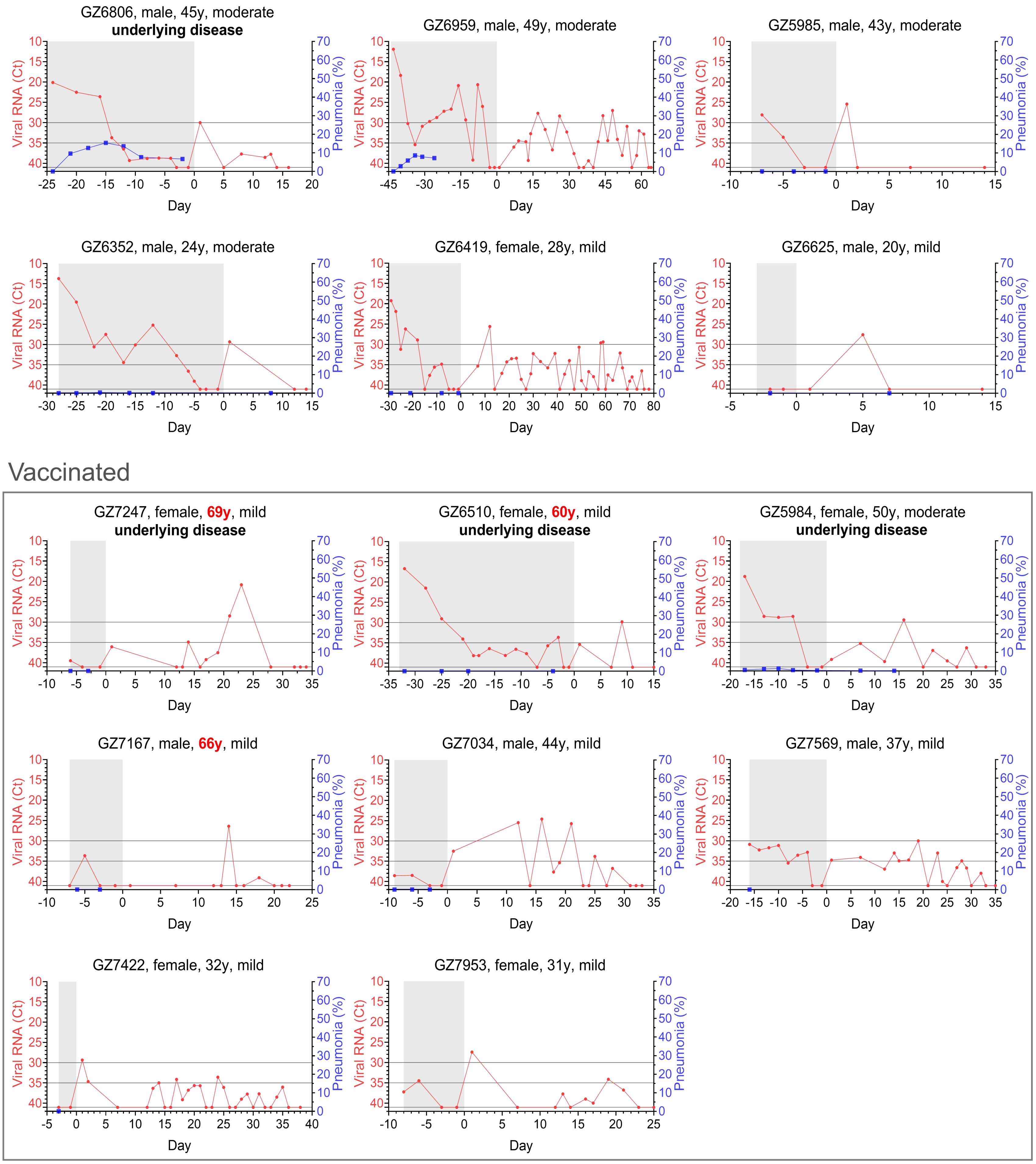

Supplementary Figure 5. Kinetics of viral RNA titers and pneumonia in 14 imported patients with a high retested viral RNA (at least one Ct<30) during quarantine. Patient number, gender, age, symptom, and comorbidity are labeled on the top of each individual. Red dot, viral RNA titers Ct value; blue square, pneumonia (ratio of abnormal lung lesions versus total lung volume); gray area, the first admission stage. Day 0 on the x-axis is the start time point when patients were discharged and transferred to quarantine wards.

Supp Figure 6

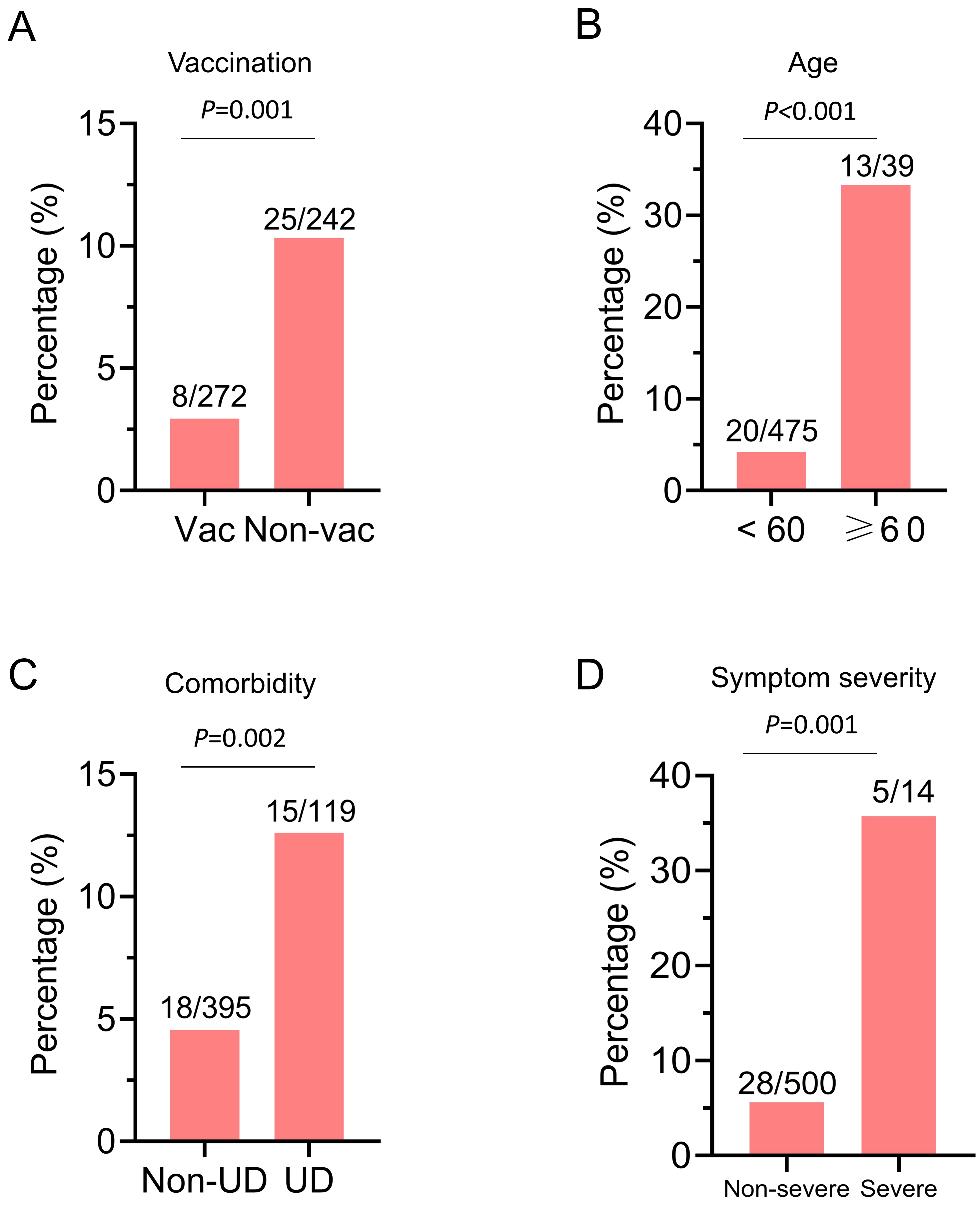

Supplementary Figure 6. Factors associated with a high level of viral RNA (Ct<30) during quarantine. (A) Vaccination status. Vac., Vaccinated patients. Non-Vac., Non-vaccinated patients. (B) Age. (C) Comorbidity. UD, patients with underlying diseases. Non-UD, patients without underlying diseases. (D) Symptom severity. *P* values were determined using the Chi-square test.

Supp Figure 7

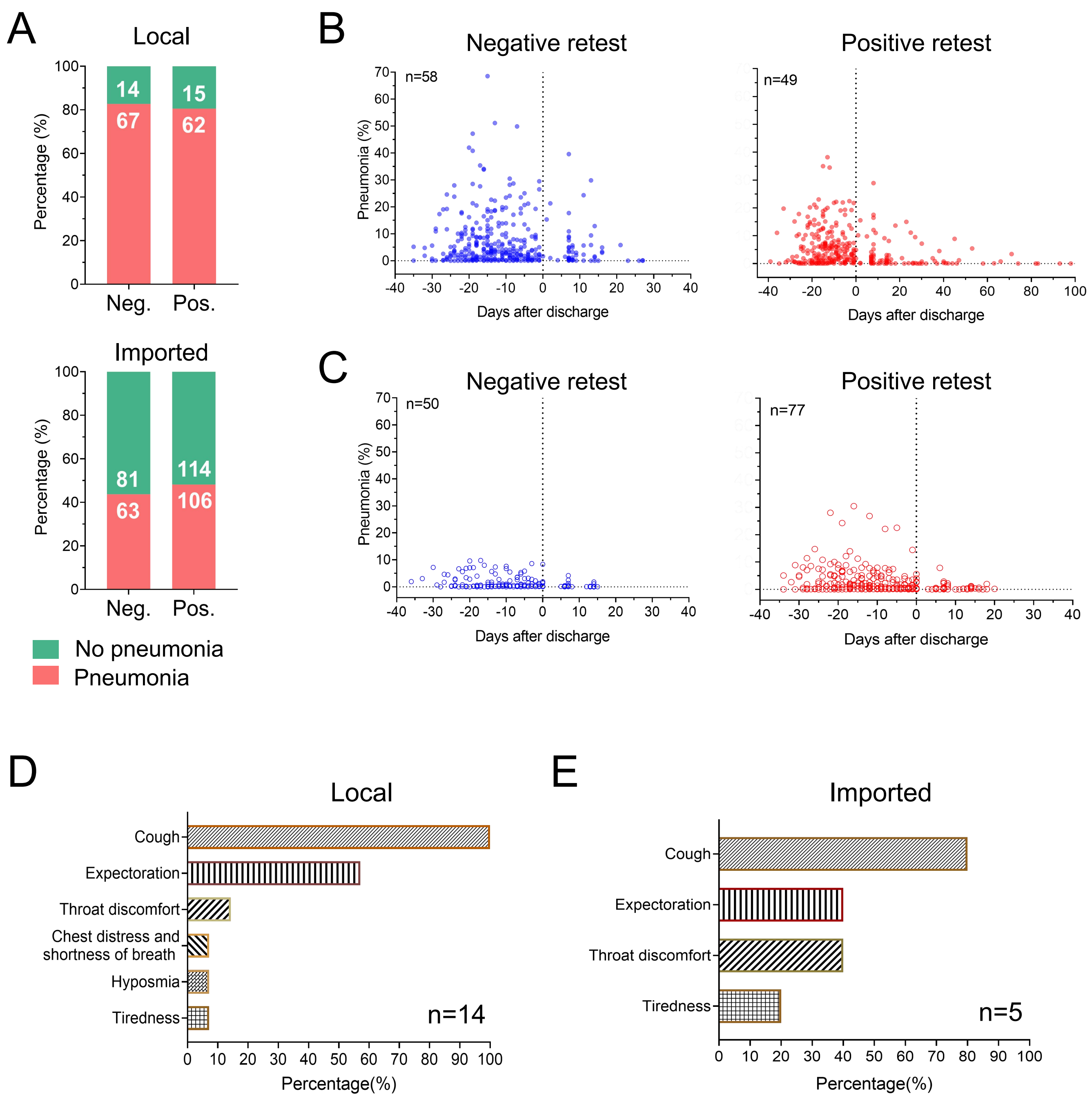

Supplementary Figure 7. Clinical characteristics of quarantined patients with positive retest viral RNA after discharge. (A) Percentage of patients with abnormal lung image (pneumonia) in positive retest patients. B and C. Kinetics of pneumonia (calculated by AI) of the total lung in local (B) and imported (C) positive retest patients from admission to the end of quarantine. D and E. Frequency of observed clinical symptoms in local (D) and imported (E) positive retest patients during quarantine.

Table. S1.Clinical characteristics of patients infected with SARS-CoV-2 Delta variant.

	Local cases (n=158)				*P*	Imported cases (n=679)				*P*
	Negative retest (n=81)		Positive retest (n=77)			Negative retest (n=242)		Positive retest (n=437)		
Age										
Median (P25-P75), yr	51	(27-64)	45	(32-71)	0.660	34	(28-47)	35	(28-44)	0.595
≤14, n(%)	10	(12.35)	9	(11.69)		3	(1.24)	3	(0.69)	
14<age≤60, n(%)	44	(54.32)	40	(51.95)	0.708	237	(97.93)	423	(96.80)	0.093
>60, n(%)	27	(33.33)	28	(36.36)		2	(0.83)	11	(2.52)	
Gender, n(%)										
Male	33	(40.74)	31	(40.26)	0.951	193	(79.75)	336	(76.89)	0.389
Female	48	(59.26)	46	(59.74)		49	(20.25)	101	(23.11)	
BMI, median (P25-P75)	22.87	(20.81- 24.34)	21.72	(19.31, 24.03)	0.164	23.42	(20.76, 25.65)	23.44	(20.76- 25.93)	0.691
Classification, n(%)										
Mild	22	(27.16)	19	(24.68)	0.329	146	(60.33)	274	(62.70)	0.522
Moderate	52	(64.20)	46	(59.74)		94	(38.84)	161	(36.84)	
Severe	4	(4.94)	3	(3.90)		2	(0.83)	2	(0.46)	
Critical	3	(3.70)	9	(11.69)		0	(0.00)	0	(0.00)	
Comorbidity, n(%)										
Hypertension	13	(16.05)	17	(22.08)	0.334	9	(3.72)	31	(7.09)	0.074
Coronary heart disease	3	(3.70)	1	(1.30)	0.649	2	(0.83)	2	(0.46)	0.938
Diabetes	6	(7.41)	7	(9.09)	0.700	3	(1.24)	15	(3.43)	0.088
Pulmonary disease	3	(3.70)	3	(3.90)	>0.999	13	(5.37)	12	(2.75)	0.082
Hepatic disease	1	(1.23)	1	(1.30)	>0.999	14	(5.79)	24	(5.49)	0.874
Kidney disease	2	(2.47)	1	(1.30)	>0.999	9	(3.72)	7	(1.60)	0.082
Mental disease	0	(0)	1	(1.30)	-	0	(0)	1	(0.23)	-
Thyroid disease	3	(3.70)	4	(5.19)	0.945	0	(0)	1	(0.23)	-

Data are presented as median (P25-P75) or number (%). *P* values were determined using the Mann-Whitney U-test for continuous variables and Chi-square test for categorical variables.

Table. S2. Laboratory results of patiensts on admission

Laboratory results	Normal Range	Local (n=158)			Imported (n=244)		
		Negative retest (n=81)	Positive retest (n=77)	*P*	Negative retest (n=119)	Positive retest (n=125)	*P*
Leukocytes, 10^9/L	3.50–9.50	5.72(4.78-6.64)	5.77(4.16-6.97)	0.530	6.38±1.83	5.69±1.72	**0.003**
Neutrophils, 10^9/L	1.80–6.30	3.78(3.02-4.77)	4.02(2.62-5.10)	0.943	3.55±1.83	3.30±1.31	0.134
Lymphocytes, 10^9/L	1.10–3.20	1.14(0.88-1.54)	1.07(0.80-1.33)	0.108	2.16±0.80	1.81±0.72	**<0.001**
Monocytes, 10^9/L	0.10–0.60	0.47(0.40-0.62)	0.51(0.36-0.68)	0.554	0.46（0.38-0.57）	0.43（0.32-0.55）	0.097
Basophils, 10^9/L	0-0.06	0.01(0.01-0.02)	0.01(0.01-0.02)	0.811	0.03（0.02-0.04）	0.02（0.01-0.04）	**0.013**
Eosinophils, 10^9/LL	0.02-0.52	0.02(0-0.06)	0.01(0-0.04)	0.255	0.12（0.06-0.19）	0.08（0.02-0.16）	**0.003**
Hemoglobin, g/L	130-175	135.23±14.99	134.69±14.08	0.814	148.42±17.57	149.98±16.27	0.471
Platelet, 10^9/L	125–350	192.00(158.50-230.50)	187.00(147.00-245.00)	0.688	216.76±60.62	214.10±61.95	0.734
serum amyloid A, mg/L	<10	12.37(6.61-69.30)	17.55(6.54-63.18)	0.756	5.96（4.70-8.95）	7.81（4.85-22.75）	**0.003**
ALT, U/L	9-50	14.50(9.70-22.00)	14.90(10.60-20.75)	0.920	16.75（10.58-36.83）	19.50（13.50-32.10）	0.447
AST, U/L	15-40	20.80(15.85-29.65)	20.65(16.20-26.00)	0.698	17.85（14.10-23.75）	19.70（15.65-21.15）	0.061
Total bilirubin, μmol/L	0-26	6.83(4.50-9.05)	6.57(4.31-8.09)	0.466	9.78（6.88-13.75）	9.17（6.68-12.61）	0.225
Albumin, g/L	40-55	43.05(40.58-46.53)	43.50(39.80-45.95)	0.772	44.60（42.68-46.33）	44.80（43.00-46.55）	0.715
Creatinine, μmol/L	57-111	69.41±23.41	68.66±24.43	0.852	74.25（64.23-83.53）	71.90（62.95-82.45）	0.452
Cystatin C, mg/L	0.59-1.03	1.02±0.27	1.03±0.33	0.693	0.96（0.88-1.05）	0.97（0.87-1.06）	0.932
LDH, U/L	120–250	209.00(171.00-249.00)	197.00(173.50-238.00)	0.761	163.00（144.00-177.00）	175.00（156.25-195.50）	**0.006**
Creatine kinase, U/L	50-310	104.00(73.00-159.00)	99.00(79.00-154.50)	0.941	69.50（59.00-124.00）	80.50（58.75-105.50）	0.482
Creatine kinase-MB, U/L	0-24	13.75(11.40-19.48)	13.70(11.50-17.95)	0.791	12.60（10.25-15.30）	13.15（10.40-15.65）	0.768
Prothrombin time, s	9.2-15	12.77(12.22-13.40)	12.95(12.58-13.72)	0.093	13.26±1.04	13.26±0.78	0.974
PTA, %	70-130	85.09±8.05	83.12±8.14	0.129	81.93±8.96	81.48±6.78	0.666
Procalcitonin, ng/ml	<0.05	0.07(0.05-0.10)	0.07(0.06-0.10)	0.070	0.04（0.03-0.06）	0.05（0.03-0.08）	0.186

Data are presented as mean±SD or median with percentiles (P25, P75). *P* values were determined using the Student's t test or Mann-Whitney U test. Neg., negative retest cases; Pos., positive retest cases; ALT, alanine aminotransferase; AST, aspartate aminotransferase; LDH, lactate dehydrogenase; PTA, prothrombin time activity.
